# Diagnostic Test Accuracy of Urine C-peptide Creatinine Ratio for the Correct Identification of the Type of Diabetes: A Systematic Review

**DOI:** 10.17925/EE.2022.18.1.2

**Published:** 2022-05-23

**Authors:** Joseph M Pappachan, Bhuvana Sunil, Cornelius J Fernandez, Ian M Lahart, Ambika P Ashraf

**Affiliations:** 1. Department of Endocrinology & Metabolism, Lancashire Teaching Hospitals NHS Trust, Preston, UK; 2. Faculty of Science, Manchester Metropolitan University, Manchester, UK; 3. Faculty of Biology, Medicine and Health, The University of Manchester, Manchester, UK; 4. Division of Pediatric Endocrinology & Diabetes, Mary Bridge Children's Hospital, Tacoma, WA, USA; 5. Department of Endocrinology & Metabolism, Pilgrim Hospital, Boston, UK; 6. Faculty of Education, Health and Wellbeing, University of Wolverhampton, Walsall, UK; 7. Division of Pediatric Endocrinology & Diabetes, University of Alabama at Birmingham, AL USA

**Keywords:** C-peptide, urine C-peptide creatinine ratio (UCPCR), endogenous insulin reserve, type 1 diabetes mellitus (T1DM), type 2 diabetes mellitus (T2DM), monogenic diabetes, maturity-onset diabetes of the young (MODY)

## Abstract

**Objective**: To examine the accuracy of urine c-peptide creatinine ratio (UCPCR) for identifying the type of diabetes in appropriate clinical settings. **Design**: Systematic review of test accuracy studies on patients with different forms of diabetes. **Data sources**: Medline, Embase and Cochrane library databases from 1 January 2000 to 15 November 2020. **Eligibility criteria**: Studies reporting the use of UCPCR for diagnosing patients with type 1 diabetes mellitus (T1DM), type 2 diabetes mellitus (T2DM) and monogenic forms of diabetes (categorized as maturity-onset diabetes of the young [MODY]). **Study selection and data synthesis**: Two reviewers independently assessed articles for inclusion and assessed the methodological quality of the studies using the Quality Assessment of Diagnostic Accuracy Studies-2 tool, with input from a third reviewer to reach consensus when there was a dispute. Meta-analysis was performed with the studies reporting complete data to derive the pooled sensitivity, specificity and diagnostic odds ratio (DOR), and narrative synthesis only for those with incomplete data. **Results**: Nine studies with 4,488 patients were included in the qualitative synthesis, while only four of these (915 patients) had complete data and were included in the quantitative synthesis. All the studies had moderate risk of bias and applicability concerns. Meta-analysis of three studies (n=130) revealed sensitivity, specificity and DOR of 84.4% (95% confidence interval [CI] 68.1–93.2%), 91.6% (82.8–96.1%) and 59.9 (32.8–106.0), respectively, for diagnosing T1DM using a UCPCR cut-off of <0.2 nmol/mmol. For participants with T2DM (three studies; n=739), UCPCR >0.2 nmol/mmol was associated with sensitivity, specificity and DOR of 92.8% (84.2–96.9%), 81.6% (61.3–92.5%) and 56.9 (31.3–103.5), respectively. For patients with MODY in the appropriate clinical setting, a UCPCR cut-off of >0.2 nmol/mmol showed sensitivity, specificity and DOR of 85.2% (73.1–92.4%), 98.0% (92.4–99.5%) and 281.8 (57.5–1,379.7), respectively. **Conclusions**: Based on studies with moderate risk of bias and applicability concerns, UCPCR confers moderate to high sensitivity, specificity, and DOR for correctly identifying T1DM, T2DM and monogenic diabetes in appropriate clinical settings. Large multinational studies with multi-ethnic participation among different age groups are necessary before this test can be routinely used in clinical practice. **Study registration**: Protocol was registered as PROSPERO CRD42017060633.

Identifying the type of diabetes correctly can be difficult, especially in adults, because of the heterogeneity in the clinical presentation. However, it is important to accurately diagnose the type of diabetes for its clinical, prognostic, therapeutic and psychosocial implications. The clinical characteristics, diagnostic work-up, therapies and complications of each form of diabetes are different, and therefore, physicians should accurately characterize diabetes after diagnosis.

Proinsulin is formed by the human pancreatic β cells, which is cleaved into insulin and C-peptide in equimolar quantities.^1^ Therefore, plasma levels of C-peptide can be used as a surrogate marker of endogenous insulin secretory capacity.^2^ Type 1 diabetes mellitus (T1DM) results from autoimmune destruction of the β cells, leading to absolute deficiency of insulin within a few years of disease onset, resulting in very low or unmeasurable plasma insulin and C-peptide levels. Conversely, in type 2 diabetes mellitus (T2DM), insulin deficiency is relative rather than absolute. Monogenic diabetes is clinically heterogeneous, but is typically an autosomal-dominant, non-insulin-dependent diabetes lacking autoantibodies, also known as maturity-onset diabetes of the young (MODY).^3^ In all types except T1DM, plasma C-peptide will be detectable. Therefore, assessing plasma C-peptide levels can be useful in both identifying diabetes subtypes and planning management strategies.^3^

However, there are practical difficulties in measuring plasma C-peptide levels, mainly the requirement that specimens be kept in ice because of the short biological half-life (30 minutes).^3^ As such, collection and transport of samples between outpatient clinics, home settings and testing laboratories is challenging.

C-peptide is largely metabolized by the kidney through glomerular filtration and tubular uptake, with only 5% of the total amount produced excreted in the urine.^4^ Urinary C-peptide remains stable at room temperature for up to 72 hours when preserved in boric acid, and can be easily transported to testing laboratories, even from remote settings.^3^ Therefore, urine C-peptide becomes an attractive and non-invasive alternative for testing β-cell function and insulin production capacity. To avoid having to collect urine for 24 hours to estimate β-cell reserve, spot urine C-peptide creatinine ratio (UCPCR) is an easy alternative option.^5^ By using modern, high-sensitivity immunoassay technologies, an Exeter research group later popularized UCPCR to measure C-peptide down to picomolar concentrations. However, the sensitivity, specificity and predictive value of UCPCR for identifying the type of diabetes is highly variable in these published reports, reducing its clinical application, especially when disease duration is considered.^6–10^ Therefore, the aim of this diagnostic test accuracy (DTA) systematic review was to critically appraise the use of UCPCR in correctly identifying the type of diabetes.

## Methods

We performed the study as per the guidelines specified in the Preferred Reporting Items for Systematic Reviews and Meta-Analyses (PRISMA).^11^ We searched the Medline, EMBASE and Cochrane Library databases to obtain articles from 1 January 2000 to 15 November 2020 (detailed search strategy is shown in *Appendix 1*). Search was limited to articles published in English, and the references of relevant studies were also searched for inclusion if relevant. Overlapping or potential duplicate data were carefully excluded using EndNote Version X8 (Clarivate™, London, UK).

### Eligibility criteria

We included a study if: (1) UCPCR was used as a diagnostic test for subtyping diabetes, and (2) positive antibody testing was used to confirm the diagnosis in patients with T1DM, and candidate genes were genetically confirmed for monogenic diabetes (MODY). A UCPCR value <0.2 nmol/mol was considered evidence of severe insulin deficiency. The gold-standard criteria used for diagnosing T1DM were continuous insulin treatment (exogenous insulin dependency) within 3 years of diagnosis, and absolute insulin deficiency (UCPCR <0.2 nmol/mmol ≥5 years post-diagnosis).^12^ Diagnosis of MODY was based on the International Society for Paediatric and Adolescent Diabetes (ISPAD) criteria: a family history of diabetes in one parent and a first-degree relative of that parent; lack of characteristics of T1DM (lack of islet-cell antibodies, low or no insulin requirement 5 years after diagnosis ± serum C-peptide levels >200 pmol/L); and lack of classical features of T2DM (marked obesity or acanthosis nigricans).^13^ The remainder were considered to be diagnosed with T2DM.

### Data extraction and quality assessment

Two independent investigators (JMP and BS) independently assessed all the titles and abstracts to identify studies eligible for inclusion in the review. The same authors then reviewed the full texts of these studies for eligibility to be included in the DTA study, and extracted data from the eligible studies using a standardized data-extraction form. The form included the following characteristics of each trial: first author's name; year of publication; study population characteristics, including sample size, geographical location, mean age and sex; and diagnostic criteria, including screening and confirmatory tests for the type of diabetes. Differences between reviewers were resolved by discussion with the third reviewer (APA).

The risk of bias and applicability of the identified studies were assessed by two independent reviewers (JMP and CJF) using the modified Quality Assessment of Diagnostic Accuracy Studies-2 (QUADAS-2) criteria, for patient selection, performance of the index test, performance of the reference test, and flow and timing.^14^ Conflicts were resolved by consensus between the two reviewers and, when necessary, with additional input from a third reviewer (APA).

### Statistical analysis and data synthesis

In our quantitative analysis, we included only studies reporting full data of true positives, true negatives, false positives and false negatives, or data obtained from eligible study publications or from authors directly. Other studies were used for qualitative synthesis only. Meta analysis was performed only if the absolute numbers of true-positive, true-negative, false-positive and false-negative results were provided or could be derived from at least two of the studies included.

The DTA measures are reported as point estimates with 95% confidence intervals (CIs). We derived the sensitivity, specificity and likelihood ratios (positive likelihood ratio [+LR] and negative likelihood ratio [-LR]) based on the true-positive, true-negative, false-positive and false-negative rates from each trial.^15^ Summary sensitivity, specificity, +LRs, -LRs and diagnostic odds ratios (DORs) also were derived using a bivariate random-effects model.

To visualize heterogeneity of diagnostic accuracy among the included studies, we plotted sensitivity and specificity of our index tests on coupled forest plots using RevMan version 5.4 (Cochrane Training, London, UK).^16^ When meta analysis was appropriate (given the number of studies and extent of clinical heterogeneity), we pooled results from the included studies. Because our random-effects meta analysis was performed for a single threshold, we chose a bivariate model for binary results to determine summary estimates of sensitivity and specificity with 95% confidence and prediction regions.^17^ All meta-analyses were performed using MetaDTA version 2.01, an online application that uses statistical software R and the existing packages Shiny and lme4.^18–20^

## Results

### Study characteristics

**Figure 1:**
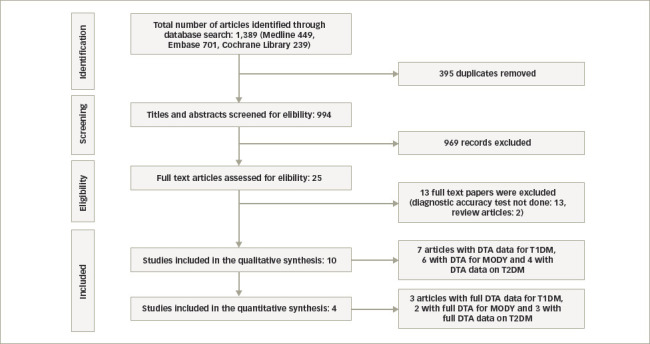
Study flow diagram

Our search on 15 November 2020 identified 1,389 citations in all three databases and, after removing duplicates, 994 titles were screened for eligibility for inclusion. The study flow diagram is shown in *Figure 1*. Of these, four studies reported the role of UCPCR among 836 patients with T2DM;^18,21–23^ seven studies studied that in 3,395 patients with T1DM;^9,10,18,24–27^ and six studies studied the role of UCPCR among 257 patients with MODY (*Table 1*).^9,10,21–28^

**Figure 2:**
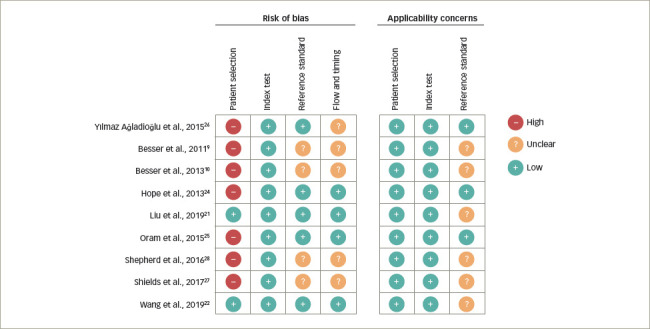
The risk of bias and applicability concerns summary: Authors' judgements about each domain for each included study^9,10,21,22,24–28^

**Figure 3:**
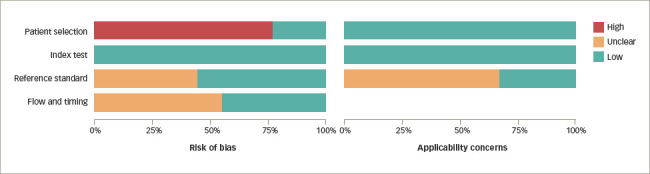
The risk of bias and applicability concerns graph: Authors' judgements about each domain for each included study

The assessment of risk of bias and applicability via QUADAS-2 is presented in *Figure 2* and *Figure 3*.^9,10,21,22,24–28^ Overall, the risk of bias was scored as ‘low’ or ‘concern’ in 100% of studies for the index test, ‘unclear’ in 42% for the reference standard test, and ‘high’ or ‘concern’ in 55% for flow and timing and 80% in the patient selection domain. The applicability of studies was scored as ‘low concern’ in 100% for patient selection and index test, and ‘unclear’ in 35% for reference standard test.

Only four studies reported (or authors shared) the full data for appropriate quantitative synthesis; therefore, complete meta-analysis could be performed.^21–24,26^ The study by Yılmaz Ağladioğlu et al. included adolescents and children aged <20 years with diabetes duration ≥2 years.^26^ In the study by Hope et al., patients with T2DM had a median duration of disease of 13 years (interquartile range [IQR] 9–17 years) and a median age of 58 years (IQR 50–66 years).^24^ In the study by Liu et al., the mean age was 42.9 ± 18.5 years for patients with T1DM (age at diagnosis: 31.8 ± 17.2 years) and 56.2 ± 13.4 years for patients with T2DM (age at diagnosis: 44.1 ± 11.5 years).^21^ In the Wang et al. study, median duration of diabetes in patients with T1DM, monogenic diabetes and T2DM was 6.5 years (IQR 1.5–13.0 years), 5.0 years (IQR 1.0–16.0 years) and 8.0 years (IQR 2.3–13.0 years), respectively;^22^ the median age of patients was 46.0 years (IQR 26.5–59.5 years), 35.0 years (IQR 30.0–47.0 years) and 53.0 years (IQR 42.0–60.0 years), respectively.^22^

*DTA = Data Transfer Agreement; MODY = maturity-onset diabetes of the young; T1DM = type 1 diabetes mellitus; T2DM = type 2 diabetes mellitus.*

**Table 1:** Characteristics of individual studies included in the review^9,10,21–28^

Study, year, country	N	Index test (UCPCR cut-off in nmol/mol)	Sensitivity, %	Specificity, %	PPV, %	NPV, %
Genetic testing for MODY and exclusion of T1DM by antibody testing						
Shields et al, 2017, UK^27^	51	>0.2	98.0	85.0	20.0	99.9
Shepherd et al, 2016, UK^28^	20	>0.2	99.0	N/A	N/A	N/A
Yılmaz Ağladioğlu et al, 2015, Turkey^26^	27	>0.2	96.3	85.7	81.3	97.9
Besser et al, 2013, UK^10^	39	>0.7	100.0	97.0	N/A	N/A
Besser et al, 2011, UK^9^	97	>0.2	97.0	96.0	N/A	N/A
Wang et al, 2019, China^22^	23	>0.2	87.0	92.7	83.3	94.6
Positive T1DM by antibody tests and exclusion of MODY by genetic testing						
Shields et al, 2017, UK^27^	1,362	<0.2	N/A	N/A	N/A	N/A
Shepherd et al, 2016, UK^28^	817	<0.2	N/A	N/A	N/A	N/A
Yılmaz Ağladioğlu et al, 2015, Turkey^26^	42	<0.2	85.7	96.3	97.3	81.3
Oram et al, 2015, UK^25^	924	<0.2	N/A	N/A	N/A	N/A
Besser et al, 2013, UK^10^	120	<0.7	100.0	97.0	N/A	N/A
Liu et al, 2019, China^21^	61	<0.2	68.9	95.8	67.7	96.0
Besser et al, 2011, UK^9^	69	<0.2	N/A	N/A	N/A	N/A
90 minutes post-mixed-meal serum C-peptide (if UCPCR <0.2 nmol/mmol) and exclusion of T1DM by antibody testing						
Hope et al, 2013, UK^24^	188	>0.2	96.0	100.0	100.0	27.0
Hope et al, 2016, UK^23^	41	<0.2	83.0	93.0	90.0	N/A
WHO criteria for T2DM diagnosis and exclusion of T1DM by antibody testing						
Liu et al, 2019, China^21^	471	>0.2	95.8	68.9	96.0	67.7
WHO diagnostic criteria for T2DM, exclusion of T1DM by antibody testing and exclusion of MODY by genetic testing						
Wang et al, 2019, China^22^	136	>0.2	81.6	92.9	96.5	67.5

*MODY = maturity-onset diabetes of the young; N/A = not available; N = number; NPV = negative predictive value; PPV = positive predictive value; T1DM = type 1 diabetes mellitus; T2DM = type 2 diabetes mellitus; UCPCR = urine C-peptide creatinine ratio; WHO = World Health Organization.*

### Diagnostic value of urine C-peptide creatinine ratio for diagnosing type 1 diabetes mellitus

**Figure 4:**
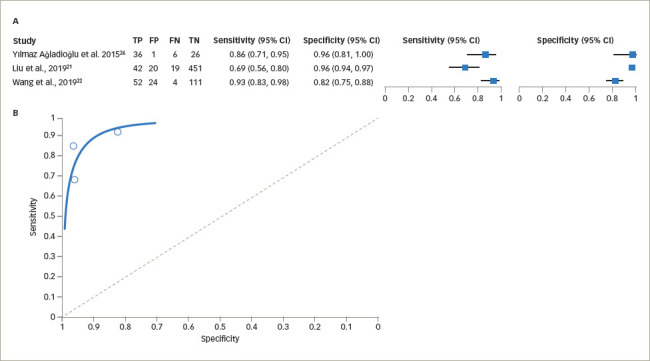
(A) Forest plot for sensitivity and specificity of urine C-peptide creatinine ratio in type 1 diabetes mellitus. (B) Summary receiver operating characteristic curve for sensitivity and specificity of urine C-peptide creatinine ratio in type 1 diabetes mellitus^21,22,26^

The sensitivity of UCPCR for diagnosing T1DM varied from 82% (95% CI 74–88%) to 96% (95% CI 92–98%) using the cut-off value of <0.2 nmol/mmol, while the specificity was 69% (95% CI 56–80%) to 100% (95% CI 29–100%). The pooled estimate of sensitivity was 84.4% (95% CI 68.1–93.2%) and the pooled estimate of specificity was 91.6% (95% CI 82.8–96.1%; *Figure 4*).^21,22,26^ The pooled estimate of false-positive rate was 8.4% (95% CI 3.9–17.2%). The DOR was estimated as 59.9 (95% CI 32.8–106.0). The +LR was 10.1 (95% CI 5.3–19.2) and the -LR was 0.17 (95% CI 0.08–0.36).

Full data on the sensitivity, specificity, positive predictive value (PPV) and negative predictive value (NPV) were not available from four studies,^9,25,27,28^ and the study by Besser et al. in 2013 used a UCPCR cut-off of <0.7 nmol/mol; therefore, we could not include these studies in the meta-analysis.^10^ The available data on these studies are depicted in *Table 1*.

### Diagnostic value of urine C-peptide creatinine ratio for diagnosing type 2 diabetes mellitus

**Figure 5:**
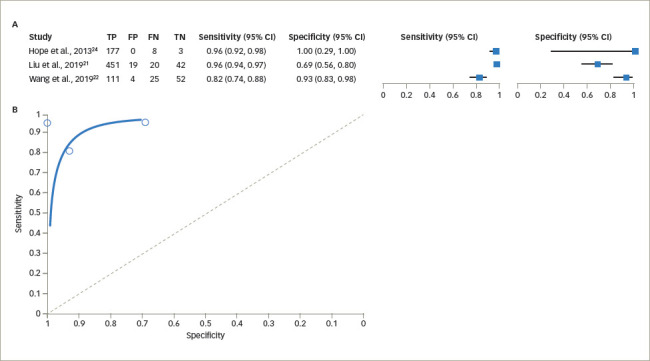
(A) Forest plot for sensitivity and specificity of urine C-peptide creatinine ratio in type 2 diabetes mellitus. (B) Summary receiver operating characteristic curve for sensitivity and specificity of urine C-peptide creatinine ratio in type 2 diabetes mellitus^21,22,24^

The reported sensitivity of UCPCR for diagnosing T2DM varied from 81.6% to 96.0% keeping the cut-off value of >0.2 nmol/mol, while the specificity was 68.9–100%. The pooled estimate of sensitivity was 92.8% (95% CI 84.2–96.9%) and the pooled estimate of specificity was 81.6% (95% CI 61.3–92.5%; *Figure 5*)^21,22,24^ The pooled estimate of falsepositive rate was 18.4% (95% CI 7.5–38.7%). The DOR was estimated as 56.9 (95% CI 31.3–103.5). The +LR was 5.04 (95% CI 2.3–11.1) and the -LR was 0.09 (95% CI 0.05–0.17).

*CI = confidence interval; FN = false negative; FP = false positive; TN = true negative; TP = true positive.*

*CI = confidence interval; FN = false negative; FP = false positive; TN = true negative; TP = true positive.*

**Table 2:** Sensitivity analysis for the studies included in the quantitative synthesis^21,22,24,26^

T1DM sensitivity											
Yılmaz Ağladioğlu^26^ and Liu^21^	Yılmaz Ağladioğlu^26^ and Wang^22^	Liu^21^ and Wang^22^
			Parameter	Estimate	2.5% CI	97.5% CI	Parameter	Estimate	2.5% CI	97.5% CI	Parameter	Estimate	2.5% CI	97.5% CI
			Sensitivity	0.771	0.628	0.870	Sensitivity	0.898	0.800	0.951	Sensitivity	0.840	0.585	0.951
			Specificity	0.958	0.927	0.976	Specificity	0.884	0.698	0.962	Specificity	0.912	0.773	0.969
False-positive rate	0.042	0.024	0.073	False-positive rate	0.116	0.038	0.302	False-positive rate	0.088	0.031	0.227
Random-effects correlation	1	N/A	N/A	Random-effects correlation	-1	N/A	N/A	Random-effects correlation	-1	N/A	N/A
logit(sensitivity)	1.211	0.523	1.899	logit(sensitivity)	2.176	1.387	2.965	logit(sensitivity)	1.659	0.345	2.973
logit(specificity)	3.135	2.548	3.722	logit(specificity)	2.033	0.836	3.230	logit(specificity)	2.336	1.224	3.448
T2DM sensitivity											
Hope (2013)^24^ and Liu^21^				Hope (2013)^24^ and Wang ^22^				Liu^21^ and Wang ^22^			
Parameter	Estimate	2.5% CI	97.5% CI	Parameter	Estimate	2.5% CI	97.5% CI	Parameter	Estimate	2.5% CI	97.5% CI
Sensitivity	0.957	0.939	0.970	Sensitivity	0.907	0.759	0.968	Sensitivity	0.910	0.764	0.969
Specificity	0.703	0.581	0.802	Specificity	0.957	0.555	0.997	Specificity	0.840	0.585	0.951
False-positive rate	0.297	0.198	0.419	False-positive rate	0.043	0.003	0.445	False-positive rate	0.160	0.049	0.415
Random-effects correlation	NaN	N/A	N/A	Random-effects correlation	1	N/A	N/A	Random-effects correlation	-1	N/A	N/A
logit(sensitivity)	3.110	2.732	3.489	logit(sensitivity)	2.280	1.147	3.412	logit(sensitivity)	2.315	1.175	3.455
logit(specificity)	0.862	0.326	1.398	logit(specificity)	3.101	0.221	5.981	logit(specificity)	1.660	0.345	2.974

*CI = confidence interval; N/A = not applicable; NaN = not a number; T1DM = type 1 diabetes mellitus; T2DM = type 2 diabetes mellitus.*

A sensitivity analysis for studies with patients with T1DM or T2DM is shown in *Table 2*.^21,22,24,26^

The study by Hope et al. reported the use of UCPCR for identifying insulin deficiency among patients with T2DM, but no other studies reported comparable data in this review and therefore could not be meta-analysed.^24^

### Diagnostic value of urine C-peptide creatinine ratio for diagnosing maturity-onset diabetes of the young

**Figure 6:**
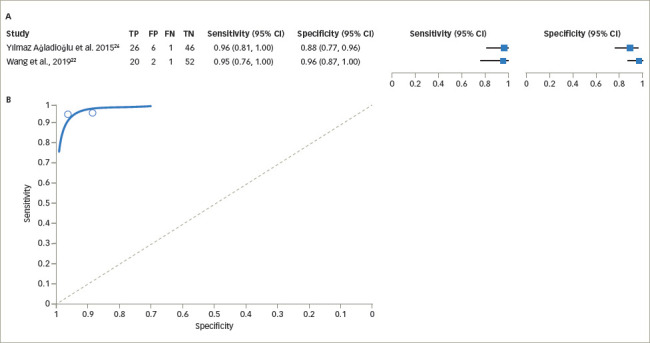
(A) Forest plot for sensitivity and specificity of urine C-peptide creatinine ratio in maturity-onset diabetes in the young. (B) Summary receiver operating characteristic curve for sensitivity and specificity of urine C-peptide creatinine ratio in maturity-onset diabetes of the young^22,26^

The reported sensitivity of UCPCR for diagnosing MODY varied from 95.0% to 96.0%, keeping the cut-off value of >0.2 nmol/mol, while the specificity was 88–96%. The pooled estimate of sensitivity was 85.2% (95% CI 73.1–92.4%) and the pooled estimate of specificity was 98.0% (95% CI 92.4–99.5%; *Figure 6*).^22,26^ The pooled estimate of false-positive rate was 2.0% (95% CI 0.5–7.6%). The DOR was estimated as 281.8 (95% CI 57.5–1,379.7). The +LR was 42.6 (95% CI 10.8–168.7) and the -LR was 0.15 (95% CI 0.08–2.87).

Full data on the sensitivity, specificity, PPV and NPV were not available from three studies, and the study by Besser et al. in 2013 used a UCPCR cut-off of >0.7 nmol/mol; therefore, we could not include these studies in the meta-analysis.^9,10,27,28^

### Discussion

#### Main findings

This systematic review assessed the use of UCPCR for correctly identifying the type of diabetes in ethnically diverse populations from the UK, China and Turkey; however, the ethnic representation of the entire study does not correctly reflect global diversity. Nonetheless, UCPCR is a relatively easy laboratory test to perform, compared with serum C-peptide assay, for identifying absolute insulin deficiency, and can be performed in diverse clinical and experimental settings to replicate the results from this study.

We found considerable heterogeneity among studies, with concerns about moderate risk of bias and applicability of the index test in the studies included in this review. Moreover, several studies did not report full data for the meta-analysis and, therefore, pooled estimates on the sensitivity, specificity and DOR could not be derived for all the studies.

The pooled estimates on the value of a UCPCR cut-off of <0.2 nmol/mol in the studies in patients with T1DM (n=130) indicate moderate sensitivity (84.4%), high specificity (91.6%) and moderate DOR (59.9) for the test in the appropriate clinical settings (as mentioned in the Methods section). Similarly, a UCPCR cut-off of >0.2 nmol/mol among participants with T2DM (n=739) indicates high sensitivity (92.8%), moderate specificity (81.6%) and moderate DOR (58.9) for correctly diagnosing T2DM. However, we have to be mindful about using the test in patients with longstanding and poorly controlled T2DM, or in those with some forms of monogenic diabetes, as insulin deficiency in these patients is not uncommon and the biological behaviour of the disease often simulates that of T1DM.^29–31^ The pooled estimates on the value of a UCPCR cut-off of >0.2 nmol/mol in participants with monogenic diabetes (n=46) show the test has moderate sensitivity (85.2%) and high specificity (98.0%) and DOR (281.8) when performed in appropriate clinical settings.

*CI = confidence interval; FN = false negative; FP = false positive; TN = true negative; TP = true positive.*

#### Limitations and the strengths of this review

Although we could procure nine different studies (seven each reporting the value of UCPCR for identifying T1DM and MODY) for the qualitative synthesis, we could obtain full data only from four studies for the meta-analysis. The total number of patients from these four studies was only 915, which is small for a common disease such as diabetes mellitus, and hampers the generalizability of the results. Moreover, lack of representation of major ethnic groups and nationalities other than Caucasian (British), Chinese and Turkish, and the under-representation of different age groups (such as the elderly or children), might limit the applicability of the results across demographics. Despite these limitations, ours is the first review examining the use of UCPCR – a simple and easily performed laboratory test – that shows moderate-to-high sensitivity, specificity and DOR for correctly identifying the main types of diabetes in an appropriate clinical setting. From a clinician's perspective, classifying the three main subtypes of diabetes can facilitate individualized treatment and thereby optimize glycaemic control, minimize risk and reduce healthcare costs.

#### Applicability of findings to the review question and conclusions

The studies evaluated in this DTA systematic review revealed moderate concerns of risk of bias and applicability among three ethnically diverse populations from the UK, China and Turkey. A UCPCR cut-off of <0.2 nmol/mol has a sensitivity of 84.4%, a specificity of 91.6% and a DOR of 59.9 for correctly identifying patients with T1DM. A UCPCR cut-off of >0.2 nmol/mol appears to confer sensitivity, specificity and DOR of 92.8%, 81.6% and 56.9, respectively, among patients with T2DM in the appropriate clinical setting. Among patients with monogenic diabetes, a UCPCR cut-off of >0.2 nmol/mol achieves a diagnostic sensitivity of 85.2%, specificity of 98.0% and DOR of 281.8 when T1DM and T2DM are excluded by appropriate clinical and biochemical profiling. Large multinational studies with demographically diverse populations are required to optimally analyse the utility of this laboratory test for routine clinical practice.

## References

[1] Steiner DF, Oyer PE (1967). The biosynthesis of insulin and a probable precursor of insulin by a human islet cell adenoma.. Proc Natl Acad Sci USA..

[2] Wentworth JM, Bediaga NG, Giles LC (2019). Beta cell function in type 1 diabetes determined from clinical and fasting biochemical variables.. Diabetologia..

[3] Jones AG, Hattersley AT (2013). The clinical utility of C-peptide measurement in the care of patients with diabetes.. Diabet Med..

[4] Matthews DR, Rudenski AS, Burnett MA (1985). The half-life of endogenous insulin and C-peptide in man assessed by somatostatin suppression.. Clin Endocrinol (Oxf)..

[5] Meistas MT, Rendell M, Margolis S, Kowarski AA (1982). Estimation of the secretion rate of insulin from the urinary excretion rate of C-peptide. Study in obese and diabetic subjects.. Diabetes..

[6] Bowman P, McDonald TJ, Shields BM (2012). Validation of a single-sample urinary C-peptide creatinine ratio as a reproducible alternative to serum C-peptide in patients with type 2 diabetes.. Diabet Med..

[7] Jones AG, Besser RE, McDonald TJ (2011). Urine C-peptide creatinine ratio is an alternative to stimulated serum C-peptide measurement in late-onset, insulin-treated diabetes.. Diabet Med..

[8] McDonald TJ, Knight BA, Shields BM (2009). Stability and reproducibility of a single-sample urinary C-peptide/creatinine ratio and its correlation with 24-h urinary C-peptide.. Clin Chem..

[9] Besser RE, Shepherd MH, McDonald TJ (2011). Urinary C-peptide creatinine ratio is a practical outpatient tool for identifying hepatocyte nuclear factor 1-α/hepatocyte nuclear factor 4-α maturity-onset diabetes of the young from long-duration type 1 diabetes.. Diabetes Care..

[10] Besser RE, Shields BM, Hammersley SE (2013). Home urine C-peptide creatinine ratio (UCPCR) testing can identify type 2 and MODY in pediatric diabetes.. Pediatr Diabetes..

[11] Liberati A, Altman DG, Tetzlaff J (2009). The PRISMA statement for reporting systematic reviews and meta-analyses of studies that evaluate health care interventions: Explanation and elaboration.. Ann Intern Med..

[12] Whiting PF, Rutjes AW, Westwood ME (2011). QUADAS-2: A revised tool for the quality assessment of diagnostic accuracy studies.. Ann Intern Med..

[13] Hope SV, Wienand-Barnett S, Shepherd M (2016). Practical classification guidelines for diabetes in patients treated with insulin: A cross-sectional study of the accuracy of diabetes diagnosis.. Br J Gen Pract..

[14] Rubio-Cabezas O, Hattersley AT, Njølstad PR (2014). ISPAD Clinical Practice Consensus Guidelines 2014. The diagnosis and management of monogenic diabetes in children and adolescents.. Pediatr Diabetes..

[15] Devillé WL, Buntinx F, Bouter LM (2002). Conducting systematic reviews of diagnostic studies: didactic guidelines.. BMC Med Res Methodol..

[16] Review Manager (RevMan) [Computer program]. Version 5.4, The Cochrane Collaboration, 2020..

[17] Chu H, Cole SR (2006). Bivariate meta-analysis of sensitivity and specificity with sparse data: A generalized linear mixed model approach.. J Clin Epidemiol..

[18] MetaDTA: Diagnostic Test Accuracy Meta-analysis. MetaDTA: Diagnostic Test Accuracy Meta-Analysis v2.01 (17th August 2021).. https://crsu.shinyapps.io/dta_ma/.

[19] Patel A, Cooper NJ, Freeman SC, Sutton AJ (2021). Graphical enhancements to summary receiver operating charcateristic plots to facilitate the analysis and reporting of meta-analysis of diagnostic test accuracy data.. Res. Synth. Methods..

[20] Freeman SC, Kerby CR, Patel A (2019). Development of an interactive web-based tool to conduct and interrogate metaanalysis of diagnostic test accuracy studies: MetaDTA.. BMC Med. Res. Methodol..

[21] Liu W, Huang X, Zhang X (2019). Urinary C-peptide creatinine ratio as a non-invasive tool for identifying latent autoimmune diabetes in adults (LADA).. Diabetes Metab Syndr Obes..

[22] Wang Y, Gao Y, Cai X (2019). Clinical implications of urinary C-peptide creatinine ratio in patients with different types of diabetes.. J Diabetes Res..

[23] Hope SV, Knight BA, Shields BM (2016). Random non-fasting C-peptide: bringing robust assessment of endogenous insulin secretion to the clinic.. Diabet Med..

[24] Hope SV, Jones AG, Goodchild E (2013). Urinary C-peptide creatinine ratio detects absolute insulin deficiency in type 2 diabetes.. Diabet Med..

[25] Oram RA, McDonald TJ, Shields BM (2015). Most people with long-duration type 1 diabetes in a large population-based study are insulin microsecretors.. Diabetes Care..

[26] Yılmaz Ağladioğlu S, Sagsak E, Aycan Z (2015). Urinary C-peptide/ creatinine ratio can distinguish maturity-onset diabetes of the young from type 1 diabetes in children and adolescents: A single-center experience.. Horm Res Paediatr..

[27] Shields BM, Shepherd M, Hudson M (2017). Population-based assessment of a biomarker-based screening pathway to aid diagnosis of monogenic diabetes in young-onset patients.. Diabetes Care..

[28] Shepherd M, Shields B, Hammersley S (2016). Systematic population screening, using biomarkers and genetic testing, identifies 2.5% of the UK pediatric diabetes population with monogenic diabetes.. Diabetes Care..

[29] Hope SV, Knight BA, Shields BM (2018). Random non-fasting C-peptide testing can identify patients with insulin-treated type 2 diabetes at high risk of hypoglycaemia.. Diabetologia..

[30] Anjana RM, Pradeepa R, Unnikrishnan R (2021). New and unique clusters of type 2 diabetes identified in Indians.. J Assoc Physicians India..

[31] McCoy RG, Galindo RJ, Swarna KS (2021). Sociodemographic, clinical, and treatment-related factors associated with hyperglycemic crises among adults with type 1 or type 2 diabetes in the US from 2014 to 2020.. JAMA Netw Open..

